# Assessing factors associated with owner’s individual decision to vaccinate their dogs against rabies: A house-to-house survey in Ouagadougou, Burkina Faso

**DOI:** 10.14202/vetworld.2021.1014-1019

**Published:** 2021-04-27

**Authors:** Madi Savadogo, Abdoul-Fataf Soré, Laibané Dieudonné Dahourou, Walter Ossebi, Alima Hadjia Banyala Combari, Rianatou Bada Alambedji, Zékiba Tarnagda

**Affiliations:** 1 Laboratoire National de Référence-Grippes (LNR-G), Unité des Maladies à potentiel Epidémique, Maladies Emergentes et Zoonoses, Département de Biologie Médicale et Santé Publique, Institut de Recherche en Sciences de la Santé (IRSS/CNRST), P.O. Box 7047, Ouagadougou, Burkina Faso; 2 Service de Microbiologie, Immunologie et Pathologies Infectieuses, Département de Santé Publique et Environnement, Ecole Inter-Etats des Sciences et Médecine Vétérinaires (EISMV), P.O. Box 5077, Dakar, Dakar, Senegal; 3 Department of Animal Husbandry, Environmental Sciences and Rural Development Institute, University of Dedougou (UDDG), P.O. Box 174, Dedougou, Burkina Faso; 4 Service d’Economie Rurale et Gestion, Département des Sciences Biologiques et Productions Animales, Ecole Inter-Etats des Sciences et Médecine Vétérinaires (EISMV), P.O. Box 5077, Dakar, Dakar, Sénégal; 5 Department of Animal Production, Environment and Agricultural Research Institute (INERA/CNRST), P.O. Box 910, Bobo Dioulasso, Burkina Faso; 6 Fundamental and Applied Research for Animals and Health (FARAH), Faculty of Veterinary Medicine, University of Liege, Quartier Vallée 2 avenue de Cureghem 10, Liege, Belgium

**Keywords:** Burkina Faso, dog vaccination, household survey, owned dogs, rabies control

## Abstract

**Background and Aim:**

In rabies endemic area, dog vaccination is an effective way of controlling the disease in animals and humans if a minimum of 70% vaccination coverage is reached. This study aimed to identify dog demographics and household characteristics associated with dogs’ vaccination against rabies in Ouagadougou, Burkina Faso.

**Materials and Methods:**

A questionnaire was used to collect data from respondents with regard to their dogs’ demographics and their household characteristics. Chi-square test and Fisher’s exact test were performed to assess the association between explicative variables and the dogs’ vaccination status.

**Results:**

Overall, as per the findings of this study, it was determined that out of 424 dogs, 57.8% were reportedly vaccinated. The vaccination status was significantly associated with most of the household variables (e.g., gender of the respondent, age, level of education, main means of transportation, participation in a vaccination campaign, knowledge on rabies, and knowledge on dog vaccination) and the dogs’ variables (breed of dog, dog origin, purpose for keeping, confinement status, and perceived behavior) (p<0.05). Moreover, only religion, type of housing, knowledge of rabies transmission modes, and dog sex were not significantly associated with vaccination status (p>0.05).

**Conclusion:**

Our study generated informative data showing that animal health workers could develop effective rabies vaccination strategy planning by examining owned dog demographics and their husbandry practices in households.

## Introduction

Rabies is considered a fatal disease transmitted to humans by bites mainly of domestic carnivores. It remains a major public health threat in Burkina Faso [1-4]. In the country’s administrative capital, Ouagadougou, from 2003 to 2014, more than 60 persons have reportedly died from rabies; 40% of them were children under 15 years old [5]. The annual number of dog bites recorded in the country is one of the highest in the West Africa subregion [6-9].

To control and stop its threat, the country made rabies a notifiable disease [10]. Indeed, at the national level, regulations have prohibited dogs from roaming around and made the annual rabies vaccination compulsory for all dogs from 3 months of age and older. Most of the time, dog rabies vaccination should be possible in Ouagadougou because veterinary clinics and vaccines are more available compared to rural areas. Dog vaccination is provided by private animal health professionals at an estimated average cost of 15 euros [4]. In some areas, public animal health workers are also involved in dog vaccination against rabies. Unfortunately, large and free mass dog vaccination campaigns are not often organized in the country. This situation may significantly impede the geographical and financial accessibility of dog-owning households to vaccines and vaccination services [11-14]. A study conducted in the same site by Savadogo *et al*. [4] showed a dog vaccination coverage of 33.5%. However, many studies reported that mass vaccination campaigns achieving at least 70% of vaccination coverage are necessary in rabies-endemic areas to eradicate the disease in animals and humans [15-19].

In the study area, canine breeding practices and household socioeconomic factors that could influence the owner’s decision to allow vaccination of their dogs have not been studied before. Therefore, this study aimed to identify dog demographics and household characteristics associated with rabies vaccination of dogs in Ouagadougou, Burkina Faso.

## Materials and Methods

### Ethical approval and Informed consent

This study obtained ethics approval from the Research Ethical Committee of the Université Cheikh Anta Diop (Protocole-0322/2018/CER/UCAD). Before administration of any questionnaire, participants were informed about the background and purpose of the study, highlighting that their participation was voluntary, and that their answers would be kept confidential. Only participants who verbally agreed were interviewed.

### Study site and period

Ouagadougou is the capital city of Burkina Faso, with an estimated population of 1.5 million people [10]. Located at the center of the country, the city is composed of 12 administrative boroughs and 55 administrative sectors. Recently, about 565,600 households were recorded in Ouagadougou [20]. This study was performed from August to November 2019 in 13 administrative sectors randomly selected from 12 administrative boroughs in Ouagadougou.

### Sampling and sampling method

The target population included domestic carnivore-owning households. Using an estimated vaccination coverage of 33.5% obtained in domestic carnivores [4] and a standard error of 5% with a 95% confidence level, the minimum required sample size was 342 households, but, in total, we were able to survey 384 households. The sample size was calculated using the formula of Thrusfield as follows:

N= Z^2^ P (1 − P)/M^2^

Z value is 1.96 at the confidence level of 95%, P is the estimated vaccination coverage percentage, and M is the standard error set at 0.05.

### Data collection

As data on dog breeding and demographics were not available, the surveyed households were identified through a door-to-door approach [21]. In each household, the head of the family or any other family member who gave verbal consent was interviewed. Only people aged 18 and older were included in the study. A structured questionnaire was used to collect data through face-to-face interviews. Before the administration of the questionnaire, each participant was briefed on the study background and objectives, and verbal consent was obtained. The obtained data included participant characteristics (gender, age, level of education, religion, knowledge of rabies, and rabies vaccination), household characteristics (area of location, type of housing, pets owned, presence or absence of a dog kennel, main means of transportation, and distance to the veterinary clinic), and dog characteristics (sex, type of breed, purpose of keeping, confinement status, behavior, and vaccination status).

### Statistical analysis

The obtained data were recorded in Microsoft Excel 2016 database for processing and calculating the percentages. The association between explicative variables and the vaccination status of dogs was determined using the Rx64 3.6.1 software (The R Foundation for Statistical Computing, https://cran.r-project.org/bin/windows/base/old/3.6.1/) to perform Chi-square test and Fisher’s exact test with a 95% confidence level. For these tests, statistical significance was set at 0.05.

## Results

### Description of pet owners and their household characteristics

In this study, most of the respondents were male (56.5%) and had a secondary education level (36.3%). The surveyed households were located in both the intraurban and periurban areas. In these households, a total of 523 pets were counted, including 99 cats and 424 dogs. The total number of households owning a dog was 345 (89.8%). In terms of animal rabies vaccination status, while no cat was vaccinated, 57.8% of the dogs were reportedly vaccinated. However, the owners had a vaccination certificate for only 36.5% of the vaccinated dogs. Tables-1 and 2 show all data regarding the surveyed households and characteristics of the respondents.

**Table 1:** Characteristics of pet owners and surveyed households in Ouagadougou, Burkina Faso, 2019.

Variable	Number of observed	Frequency (%)
Sex (n=384)		
Male	217	56.5
Female	167	43.5
Age (in years) (n=384)		
18-35	146	38.0
36-50	173	45.0
51 and older	65	17.0
Level of education (n=384)		
Primary	80	20.8
Secondary	139	36.3
University	78	20.3
Illiterate	87	22.6
Religion (n=384)		
Christianity	215	55.9
Islam	142	36.9
Animism	27	7.2
Study area (n=384)		
Intraurban	263	68.5
Periurban	121	31.5
Type of housing (n=384)		
Rented house	62	16.2
Personal property	321	83.8
Owned pets in households (n=384)		
Dog	285	74.2
Cat	39	10.2
Both dog and cat	60	15.6
Owned dogs vaccination status (n=424)		
Vaccinated	245	57.8
Unvaccinated	179	42.2
Dog kennel observed in surveyed households (n=345)		
Yes	94	27.2
No	251	72.8

**Table 2:** Dog-owning households rabies vaccination adoption (owning dog being vaccinated or not) in Ouagadougou, Burkina Faso, 2019.

Variable	Number of observed	Households in which dogs were not vaccinated (%)	Households in which dogs were vaccinated (%)	p-value
Gender of the respondent (n=345)				
Male	207	97 (46.9)	110 (53.1)	0.01
Female	138	85 (61.6)	53 (38.4)	
Age of respondent (in years) (n=345)				
18-35	135	62 (45.9)	73 (54.1)	0.00
36-50	158	81 (51.3)	77 (48.7)	
51 and above	52	39 (75.0)	13 (25.0)	
Level of education (n=345)				
Primary	70	54 (77.1)	16 (22.9)	0.00
Secondary	129	72 (55.8)	57 (44.2)	
University	72	21 (29.2)	51 (70.8)	
Illiterate	74	35 (47.3)	39 (52.7)	
Religion of the respondent (n=345)				
Christianity	196	109 (55.6)	87 (44.4)	0.30
Islam	128	65 (50.8)	63 (49.2)	
Animism	21	8 (38.1)	13 (61.9)	
Study area (n=345)				
Intraurban	231	86 (37.2)	145 (62.8)	0.00
Periurban	114	96 (84.2)	18 (15.8)	
Type of housing (n=345)				
Rented house	49	29 (59.2)	20 (40.8)	0.30
Owned house	296	153 (51.7)	143 (48.3)	
Main mean of transportation used by the household (n=345)				
Car	71	21 (29.6)	50 (70.4)	0.00
Motorbike	219	117 (53.4)	102 (46.6)	
Bicycle	55	44 (80.0)	11 (20.0)	
Estimated distance between household and the closest veterinary clinic (n=345)				
<5 km	297	144 (48.5)	153 (51.5)	0.00
≥5 km	48	38 (79.2)	10 (20.8)	
Having attended to a vaccination campaign in lifetime (n=345)				
Yes	148	57 (38.5)	91 (61.5)	0.00
No	197	125 (63.5)	72 (36.5)	
Knowledge of rabies vector animals (n=345)				
One to three cited^1^	265	135 (50.9)	130 (49.1)	0.03
Four and above cited	59	30 (50.8)	29 (49.2)	
None	21	17 (81.0)	4 (19.0)	
Knowledge of disease transmission modes (n=345)				
Two and above cited^2^	149	81 (64.8)	68 (35.2)	0.80
One cited	177	93 (65.6)	84 (34.4)
None	19	8 (70.4)	11 (29.6)	
Perceived rabies level of severity both in human and animal (n=345)				
Fatal	116	88 (56.9)	28 (43.1)	0.00
Severe	138	22 (86.2)	116 (13.8)	
Benign	91	72 (55.8)	19 (44.2)	
Minimum required dog age for vaccination in Burkina Faso (n=345)				
Correct answer (three months)	141	60 (42.6)	81 (57.4)	0.00
Uncorrect answer	204	122 (59.8)	82 (40.2)	
Annual frequency of dog vaccination (n=345)				
Correct answer (1 time)	162	51 (31.5)	111 (68.5)	0.00
Uncorrect answer	183	131 (71.6)	52 (28.4)	
Perceived cost of dog vaccination (n=345)				
Affordable	103	45 (43.7)	58 (56.3)	0.02
Expensive	242	137 (56.6)	105 (43.4)	

1 Vector animals cited or importance were dog, cat, monkey, cattle, pigs, bats, and reptiles,

2 roots of transmission cited of importance were bite, scratch, and lick

### Dog vaccination practices of households

Out of the 345 dog-owning households, 47.2% reported having their dogs vaccinated. Table-3 shows the reasons given by most of the owners (52.8%) for not having their dogs vaccinated. Indeed, many owners did not vaccinate their dogs because they could not transport the dogs to the vaccination site (veterinary clinics or fixed sites for mass vaccination campaigns). Most dog owners were male (60%) and lived in an intraurban area (66.9%). As shown in Table-2, the vaccination of dogs against rabies in households was significantly more likely to be adopted by men, owners between 18 and 35 years old, owners with a secondary education level, owners who were located in an intraurban area and closer to a veterinary clinic, owners who had a car as the main means of transportation, owners who had attended a vaccination campaign at least once in their lifetime, owners who knew of at least three rabies vector animals and the requirements for the vaccination of dogs (dog of 3 months old and above, one vaccination per year), and owners who perceived the vaccination cost to be affordable (p<0.05). However, no significant association was observed between vaccination adoption by dog owners and their religion or the type of housing (rented or owned) (p>0.05).

**Table 3:** Given reasons why households did not vaccinate their dogs in Ouagadougou, Burkina Faso, 2019.

Reason	Number of households in which dogs were not vaccinated (%)
Could not transport dog to vaccination site	56 (30.8)
Unable to restrain dog for vaccination	38 (20.9)
Vaccination too expensive or no money to pay for	27 (14.8)
Thought that dog was too young	19 (10.4)
Dog being aggressive	15 (8.2)
No reason given	27 (14.8)
Total	182 (100.0)

### Characteristics and vaccination status of owned dogs

Table-4 presents the characteristics of the surveyed dogs according to their vaccination status. Male dogs were preferred over female dogs in the study area, with a male per female sex ratio of 1:0.8. However, the males were vaccinated less often than the females, that is, 56.5% versus 59.4%, respectively, but the difference in vaccination coverage between males and females was not significant (p>0.05). Out of the 424 dogs belonging to the surveyed households, 369 were local dogs, whereas the vaccinated dogs were significantly more likely to be cross-bred or exotic (p<0.05). Moreover, most dogs were purchased by their owners (56.4%) for guarding purposes (74.3%), were not confined (73.3%), and were considered obedient by their owners (87%). The vaccinated dogs were significantly more likely to be owned for guarding, purchased, confined day and night, and obedient (p<0.05).

**Table 4:** Dog characteristics according to vaccination status of the 424 dogs identified in the survey in Ouagadougou, Burkina Faso, 2019.

Variable	Number of observed	Number of unvaccinated dogs (%)	Number of vaccinated dogs (%)	p-value
Sex (n=424)				
Male	237	103 (43.5)	134 (56.5)	0.60
Female	187	76 (40.6)	111 (59.4)	
Breed of dog (n=424)				
Local	369	177 (48.0)	192 (52.0)	0.00
Crossbred	12	2 (16.7)	10 (83.3)	
Exotic	43	0 (0.0)	43 (100.0)	
The way the dog was acquired by owner (origin) (n=424)				
Purchased	239	105 (43.9)	134 (56.1)	0.00
Gift	149	56 (37.6)	93 (62.4)	
Born in household	7	7 (100.0)	0 (0.0)	
Found	29	11 (37.9)	18 (62.1)	
Purpose for keeping a dog (n=424)				
Guarding	315	143 (45.4)	172 (54.6)	0.02
Companionship	109	36 (33.0)	73 (67.0)	
Confinement status (n=424)				
Confined 24 h/24	113	86 (19.3)	27 (80.7)	0.00
Roaming	311	93 (41.2)	218 (58.8)	
Dog perceived behavior (n=424)				
Obedient	369	144 (39.0)	225 (61.0)	0.00
Aggressive	55	35 (63.6)	20 (36.4)	

## Discussion

Dogs are known worldwide to be the main source of human rabies. Moreover, studies reported that if at least 70% of dogs are vaccinated against rabies in a given area, the disease can be controlled [18,19]. Therefore, knowledge of human- and dog-related drivers that may affect dog vaccination coverage is essential for the planning and implementation of successful mass dog vaccination and rabies elimination campaigns [22,23]. Considering this, we designed this study to gain insight into the dog vaccination coverage of owned dogs in Ouagadougou, Burkina Faso, as an area known to be rabies endemic [4,5]. The dog male per female sex ratio obtained (1:0.8) was different from that reported (3.02:10) in Cameroon [24] and (1:1.15) in Nigeria [25]. The observed difference reflects the owner’s preference for males in the study area, wherein the dogs are generally kept mainly for house guarding. The percentage of confined dogs in this present study was lower than that found in the Philippines [12]. This could be explained by the fact that in terms of local dog husbandry practices, owned dogs are allowed to go out and come home freely to seek food.

In this present study, the observed dog rabies vaccination coverage was lower than that required to sustain rabies eradication in dogs [19,26]. The reasons that owners reported for having not vaccinated their dogs were similar to those of other studies [12,14,21,24]. However, some unvaccinated dog owners in Ethiopia were not able to provide reasons probably due to the fact that they consider rabies as insignificant or they are not well aware of the disease [27].

In relation to the owner’s gender, men were significantly more likely to vaccinate their dogs. This observation can be explained by the fact that in many households and given local social practices, only men can make the final decision to vaccinate their dogs in Chad [28]. We also found that the number of periurban households in which dogs were reported to be vaccinated was significantly lower. Indeed, periurban areas are characterized by vulnerable life conditions, and poverty is identified as a serious barrier to vaccine accessibility in Peru [21]. Overall, the percentage of households in which dogs were reported to be vaccinated was significantly associated with the respondent’s good level of knowledge regarding rabies and dog vaccination. As suggested in many studies, information and community awareness are key steps for a successful and effective mass dog vaccination program [4,14,29].

As found by Davlin *et al*. [12], our study showed that dog sex was not significantly associated with dog rabies vaccination status. However, fully confined dogs had a significantly higher probability of being vaccinated [30,31]. Indeed, roaming dogs are not accustomed to contacts with people and could not be easily constrained by their owners to be vaccinated and receive other veterinary care. This was confirmed by our findings which showed a significantly higher percentage of vaccination in dogs that were perceived to be obedient. Like the confinement status and the perceived behavior of the dog, the breed of the dog, the way the dog was acquired, and the purpose of the dog being owned were significantly associated with the percentage of dog vaccination. Similar results were reported by a previous study in Mexico [11].

This present study showed that the most frequent reasons cited by owners of unvaccinated dogs were the inability to restrain their dogs and the difficulty in transporting them to vaccination points, suggesting that combining the door-to-door approach and increasing the number of vaccination points could improve the accessibility to vaccines and the dog vaccination coverage in rabies-endemic countries.

## Conclusion

The fight against animal and human rabies requires ecological, epidemiological, and social evidence for all involved stakeholders and decision-makers. This study showed that although most of the owned dogs were reported to be vaccinated, the percentage of vaccination coverage was lower than that required to eliminate rabies in animals and humans. The vaccination status was significantly associated with most household- and dog-related variables. The data obtained could aid animal health workers to better understand the demographics of owned dogs and to more effectively plan and develop dog rabies vaccination strategies.

## Authors’ Contributions

MS, AS, and RBA designed and supervised study implementation. MS, AS, LDD, and AHBC collected and analyzed data. MS wrote the manuscript draft. WO, RBA, and ZT reviewed the manuscript. All authors read and approved the final manuscript.
